# Left Ventricular Myocardial and Cavity Velocity Disturbances Are Powerful Predictors of Significant Coronary Artery Stenosis

**DOI:** 10.3390/jcm11206185

**Published:** 2022-10-20

**Authors:** Ibadete Bytyçi, Liliana Alves, Oscar Alves, Carla Lopes, Gani Bajraktari, Michael Y. Henein

**Affiliations:** 1 Clinic of Cardiology, University Clinical Centre of Kosovo, 10000 Prishtina, Kosovo; 2 Institute of Public Health and Clinical Medicine, Umeå University, 90187 Umea, Sweden; 3 Department of Cardiology, The Bedfordshire Hospitals NHS Trust, Luton LU4 0DZ, UK

**Keywords:** suspected coronary artery disease, insignificant coronary artery disease, dobutamine stress echocardiography

## Abstract

Background and Aim: Dobutamine stress echocardiography (DSE) is a well-established noninvasive investigation for significant coronary artery disease (CAD). The aim of this study was to evaluate the accuracy of cardiac Doppler parameters in predicting CAD. Methods: We prospectively studied 103 consecutive patients with suspected CAD based on typical symptoms; 59 proved to have CAD, and 44 patients proved to have no-CAD (*n* = 44). All patients underwent a complete stress Doppler echocardiographic examination. Total isovolumic time (T-IVT) as a marker of cavity dyssynchrony and wall motion score index (WMSI) were also calculated. Results: At peak dobutamine stress, the compromised LV longitudinal excursion (MAPSE), systolic septal and lateral velocities (s’), and diastolic indices were more pronounced in the CAD patients compared with those without CAD, but LV dimension did not differ between groups (*p* > 0.05). The WMSI was higher and t-IVT more prolonged in patients with CAD (*p* < 0.01 for both). Similarly, the changes were more pronounced in patients with significant CAD compared with insignificant CAD. On multivariate model, Δ mean s’, OR 2.016 (1.610 to 3.190; *p* < 0.001), Δ E velocity OR 2.502 (1.179 to 1.108; *p* < 0.001), Δ t-IVT 2.206 (1.180 to 2.780; *p* < 0.001) and Δ WMSI OR 1.911 (1.401 to 3.001; *p* = 0.001) were the most powerful independent predictors of the presence of CAD, particularly when significant (>75%). Δ mean s’ < 5.0 was 85% sensitive, 89% specific with AUC 0.92. Respective values for Δ E velocity <6.0 cm/s were 82%, 90% and 0.91; for Δ t-IVT > 4.5, 78%, 77% and 0.81 and for Δ FT ≥ 150 ms, 76%, 78% and 0.84 in predicating significant CAD. WMSI ≥ 0.7 was 75% sensitive, 77% specific with AUC of 0.81 in predicting significant CAD. The accuracy of DSE was higher in significant CAD compared to insignificant CAD (80% vs. 74%; *p* = 0.03). Conclusions: Compromised LV longitudinal systolic function, lower delta E wave, prolonged t-IVT, and increased WMSI were the most powerful independent predictors of the presence and significance of CAD. These finding strengthen the role of comprehensive DSE analysis in diagnosing ischemic disturbances secondary to significant CAD.

## 1. Introduction

Exercise electrocardiogram (ECG) remains a widely used technique for managing patients with suspected or established coronary artery disease (CAD), with good prognostic value. However, it is well known for its modest accuracy because of dynamic ST-T wave changes, being impacted by factors other than myocardial ischemia [1,2]. Over the last 4 decades, dobutamine stress echocardiography (DSE) has proved to be a favored functional test for diagnosing significant CAD, over and above exercise ECG, because of its significantly higher accuracy and being more patient friendly, particularly for those with musculoskeletal problems. A meta-analysis of 55 studies with 3714 patients demonstrated high DSE sensitivity and specificity of approximately 81% and 84%, respectively [3]. The accuracy is particularly higher in patients with left main stem disease and those with multivessel CAD [4]. Indeed, studies have shown that accuracy falls 8 to 12% in patients with single-vessel CAD [5], except those with significant left main stem disease who develop generalized signs of ischemia and left ventricular cavity dilatation early on in the stress protocol.

Dobutamine stress echocardiography is unique for not only assessing CAD induced myocardial ischemic dysfunction but also myocardial hibernation in patients with ischemic cardiomyopathy [6,7]. In addition, the technique is free of radiation, in contrast with other functional tests, as well as simple in its application, with a small ultrasound probe placed on the chest without a need for large cameras [8] or claustrophobic tubes [9]. While the conventional DSE assessment of ischemia is based on the development of wall motion abnormalities at fast heart rate with respect to resting [10], other echocardiographic modalities have been used and proved to be better in objectively assessing myocardial ischemic dysfunction, including myocardial velocities [11], myocardial deformation in the form of strain and strain rate abnormalities and myocardial perfusion disturbances [12,13]. The aim of this study was to evaluate the accuracy of cardiac Doppler parameters in predicting the presence and significance of CAD.

## 2. Methods

### 2.1. Study Population

We prospectively studied 103 consecutive patients with suspected CAD who presented to the cardiology department of the (Bedfordshire) Luton and Dunstable Hospital, UK, complaining of exertional angina, with or without ST-T waves changes on exercise ECG. All patients underwent DSE using conventional protocol, having been identified as carrying intermediate risk for atherosclerosis. The intermediate risk was evaluated by traditional models for which the consideration of another noninvasive technique can help and, consequently, may influence clinical decision making [14,15].

Subsequently, and based on the results, patients underwent conventional coronary angiography, the results of which classified the patients into CAD (*n* = 59) or no-CAD (*n* = 44). In addition, according to the presence of luminal stenosis, the 59 patients were subdivided into significant CAD (*n* = 30, ≥50% coronary luminal stenosis) and nonsignificant CAD (*n* = 29, <50% stenosis, Figure S1) [16].

The exclusion criteria were prior coronary intervention, significant valvular heart disease (more than mild stenosis or regurgitation), congenital heart disease, heart failure, atrial fibrillation, hemodynamic instability or significant pulmonary disease. All echocardiographic measurements were analyzed by two independent investigators blinded to the coronary angiography results. The study was conducted in accordance with institutional policies, national legal requirements and the revised Helsinki Declaration and was approved by the Ethics Committee of Bedfordshire, UK (16/NW/0247). All patients gave verbal informed consent to participate in the study.

Data collection: Details of the clinical evaluation of chest pain and cardiovascular risk assessment including dyslipidemia, diabetes mellitus (DM), arterial hypertension (AH), smoking and family history of CAD were evaluated in all patients. The cardiovascular risk was stratified based on presence and the number of risk factors into low (0–1), moderate (2–3) and high (>3) risk for cardiovascular disease.

Echocardiographic examination: All patients underwent full echocardiographic examination at rest and at peak pharmacological stress using conventional dobutamine protocol. The echocardiograms were performed by a single cardiologist with over 25 years of experience in DSE using a Philips IE33 Echocardiograph equipped with phased array transducer. All echocardiographic examinations were performed according to the recommendations of the European Association of Echocardiography and the American Association of Echocardiography [17].

Conventional M-mode and pulsed-wave myocardial Doppler velocities were recorded at rest and peak stress (speeds of 100 and 50 mm/s, respectively). Total amplitude of long axis motion was recorded from mitral annular peak systolic excursion (MAPSE) [18]. Total amplitude of annulus motion was measured from the peak inward motion, at the end of the T wave, to peak outward motion, at the nadir of the ‘a’ wave after the P wave of the superimposed ECG [19]. Doppler left ventricular (LV) longitudinal lateral, septal and posterior segment velocities were also measured at the respective mitral annular levels, with the s’ as an antegrade velocity during systole and e’ and a’ being two retrograde velocities during early diastole and atrial systole. The average s’, e’ and a’ velocities of the three segments were calculated as the sum of the corresponding velocities divided by 3. LV cavity global dyssynchrony was assessed by measuring total isovolumic time (t-IVT) and Tei Index, as previously described [20]. Total LV filling time was measured from the onset of the E wave to the end of the A wave and ejection time from the onset to the end of the aortic Doppler flow velocity. Total isovolumic time (t-IVT) was determined as 60—(total ejection time + total filling time) and was expressed in s/min, while Tei index was calculated as the ratio between t-IVT and ejection time [21,22]. The wall motion score index (WMSI) was calculated by assigning each LV myocardial segment a score according to its systolic function (normal = 1, hypokinesis = 2, akinesis = 3, dyskinesis = 4), as previously described [23]. The WMSI was calculated by dividing the sum of the wall motion scores of all segments by 16 [24].

The stress echocardiogram was performed using dobutamine infusion and an IVAC pump with a starting dose of 10 µg/kg/min and increasing by similar incremental dose every 3 min, to a maximum dose of 40 µg/kg/min. Stress endpoints in patients were development of symptoms (chest pain, discomfort or breathlessness), a drop in systolic blood pressure by 20 mmHg, ischemic ECG changes such as ST segment shift, T wave inversion or arrhythmia. When patients failed to reach the target heart rate with maximum dobutamine dose, they were given 300 mic of atropine iv. Blood pressure (systolic and diastolic) was measured automatically at rest and at the end of each stress stage using automated sphygmomanometer (Welch Allyn Spot Vital Signs LXi, Largo, FL, USA) [22]. A 12-lead ECG and oxygen saturation were also recorded at rest and at the end of each stress stage [25] and also every minute during recovery, using a 12-lead ECG monitor (GE Healthcare MAC 5500, Wauwatosa, WI, USA).

Coronary angiography: All patients underwent conventional coronary angiographic examination based on the extent of risk factors, symptoms and stress echocardiographic findings. The Judkin’s technique was used with at least four views of the left coronary system and two views of the right coronary system. Coronary angiograms were processed off-line using raw DICOM format and were analyzed by two senior cardiologists independently.

### 2.2. Statistical Analysis

Statistical analysis was performed using SPSS Software Package version 26.0 (IBM Corp., Armonk, NY, USA). Data are summarized using frequencies (percentages) for categorical variables and mean ± standard deviation (SD) for continuous variables or median interquartile (IQR) ranges. Continuous data were compared with two-tailed Student *t* test and discrete data with chi-square test. Variables were compared between the two groups using the unpaired Student *t* test or Fisher’s exact probability test. Clinical (age, female gender, diabetes, arterial hypertension, dislipidemia and number of risk factors) and echocardiographic predictors (delta EF, delta WMSI, delta MAPSE, delta mean s’, delta E velocity, delta A velocity and delta total IVT) of the presence and significant CAD were identified using univariate analysis, and multivariate logistic regression method. The receiver operational characteristic (ROC) analyses were performed, and the best cut-off, sensitivity and specificity were determined. A significant difference was defined as *p* < 0.05 (2-tailed).

## 3. Results

### 3.1. Demographic and Clinical Indices of the Patients

The mean age of the study population was 63.3 ± 8.8 years (45.6% females). Out of 103 patients, only 39 (37.8%) had typical exertional chest pain. Some 65 (63.1%) had hypertension, 24 (23.3%) diabetes and 69 (67.1%) dyslipidemia; 51 (49.5%) smoked and 52 (50.4%) had family history of CAD. Low risk factors for cardiovascular disease were present in 29 (28.1%), moderate risk in 66 (64.1%) and high risk in 15 (14.5%) patients. Patients with CAD were older (*p* = 0.01) and less frequently females (30.5 vs. 63%; *p* = 0.001) and had higher cardiovascular risk factors compared with those with no-CAD (*p* < 0.05, for all, Table S1).

### 3.2. Left Ventricular Function in Patients with and without CAD

At rest, there were no difference in LV dimensions, ejection fraction or LV myocardial velocities between the two groups (*p* > 0.05 for all). In patients with CAD, LV longitudinal systolic function was reduced: MAPSE-l, MAPSE-s and MAPSE-*p* (*p* < 0.05 for all) compared with no-CAD. Again, in patients with CAD, markers of global LV dyssynchrony including t-IVT (t-IVT; 10.4. ± 3.2 vs. 7.29 ± 1.3; *p* = 0.01) and Tei index (0.51 ± 0.2 vs. 0.32 ± 0.2; *p* = 0.02) were worse compared with those with no CAD. Furthermore, WMSI was significantly higher in CAD compared with no-CAD patients (*p* = 0.01; Table S2).

At peak stress: LV function measurements increased in the two groups, but to a lesser extent in the CAD patients; EF (9.9 vs. 15.6%; *p* = 0.02), MAPSE-l (9.2 vs. 12.9%; *p* = 0.002), MAPSE-s (10.9 vs. 14.9%; *p* = 0.002) and MAPSE-*p* (14.7 vs. 18.3%; *p* = 0.001) compared with resting values (*p* < 0.05 for all). Similarly, LV lateral, septal and posterior systolic velocities (s’) were lower in the CAD compared with no-CAD (*p* < 0.05 for all). LV lateral and septal e’ and a’ velocities increased (*p* < 0.05 for all) equally in both groups (*p* > 0.05). In contrast, LV cavity E wave (22.8 vs. 9.1%; *p* = 0.001) and A wave velocities increased more (19.1 vs. 8.1%; *p* = 0.001) while E/e’ ratio decreased (−17.3 vs. 2.57%; *p* < 0.001) in CAD compared with no-CAD patients. Likewise, markers of global LV dyssynchrony worsened in CAD patients; t-IVT prolonged (7.30 ± 3.1 vs. 3.20 ± 1.1; *p* = 0.01) and Tei index increased (0.35 ± 0.1 vs. 0.21 ± 0.1; *p* = 0.02) compared to no-CAD patients. WMSI was significantly higher (*p* = 0.001) in CAD patients (Table 1 and Table S2).

### 3.3. Left Ventricle Function in Patients with and without Significant CAD

At rest, LV dimensions and EF were not different between patients with and without significant CAD (*p* > 0.05 for all). LV MAPSE-l, MAPSE-s and MAPSE-*p* were lower in significant CAD (*p* < 0.05 for all) compared with nonsignificant CAD. LV lateral, septal and posterior s’ tended to be lower in the significant CAD patients (*p* = 0.06, *p* = 0.07, *p* = 0.07, respectively). LV septal and posterior e’ velocities (*p* > 0.05) as well as E/a and E/e’ ratio did not differ between groups (*p* > 0.05 for both). Similarly, there was no difference in LV global dyssynchrony, in the form of t-IVT or Tei index, between groups (*p* > 0.05 for both), while WMSI was higher in the significant CAD (*p* = 0.01) compared with nonsignificant CAD patients (Table S3).

At peak stress: The Δ LV dimensions was similar in both groups (*p* > 0.05 for all). LV EF increased in the two groups (*p* < 0.05) but to a lesser extent in the significant CAD patients (7.7 vs. 13.0%; *p* = 0.04), as did systolic longitudinal function (MAPSE-l, MAPSE-s and MAPSE-*p*). In the same way, LV left, septal and posterior s’ were more compromised in the significant than in the nonsignificant CAD patients (*p* < 0.05 for all). LV lateral e’ and a’ velocities increased similarly in both groups (*p* < 0.05 for all), and E/e’ ratio was higher (10.4 vs. 1.3%; *p* < 0.001) in patients with significant CAD. Global LV markers of dyssynchrony were worse in significant CAD patients with t-IVT: −24.1 vs. −37.1%, *p* = 0.01 and Tei index: −23.5 vs. −32.6, *p* = 0.001, compared with no significant CAD. WMSI was higher (*p* = 0.01) in significant CAD patients (Table 2 and Table S3).

### 3.4. Predictors of CAD

#### Clinical Predictors

**Figure 1:**
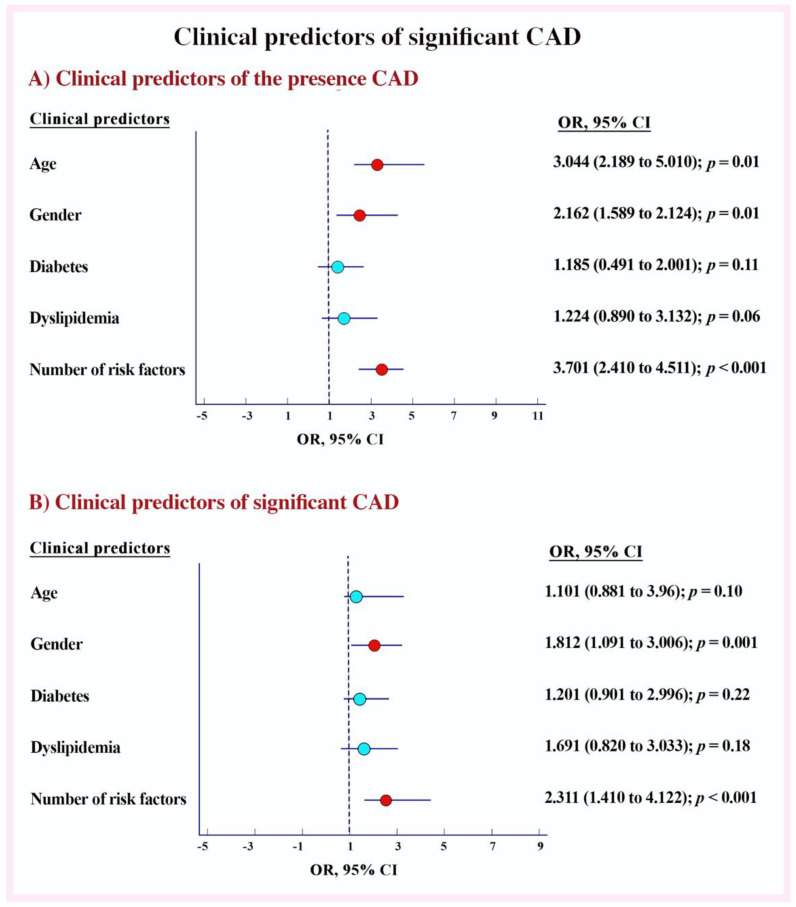
Clinical predictors of significant CAD. (**A**) Clinical predictors of the presence CAD. (**B**) Clinical predictors of significant CAD.

In univariate analysis, age (*p* = 0.002), female gender (*p* = 0.001), diabetes (*p* = 0.03), dyslipidemia (*p* = 0.001) and number of risk factors (*p* < 0.001) predicted the presence of CAD. In the multivariate model, only age (OR 3.044; 2.189 to 5.010; *p* = 0.01), female gender (OR 1.162; 1.589 to 2.124; *p* = 0.01) and number of cardiac risk factors (OR 3.701; 2.410 to 4.511; *p* < 0.001) predicted the presence of CAD (Figure 1A and Table S3). Similarly, in multivariate analysis, only the number of cardiac risk factors (OR 2.311; 1.610 to 4.122; *p* < 0.001) and female gender (OR 2.812; 1.601 to 4.006; *p* = 0.001) predicted significant CAD (Figure 1B and Table S4).

### 3.5. Echocardiographic Predictors

**Figure 2:**
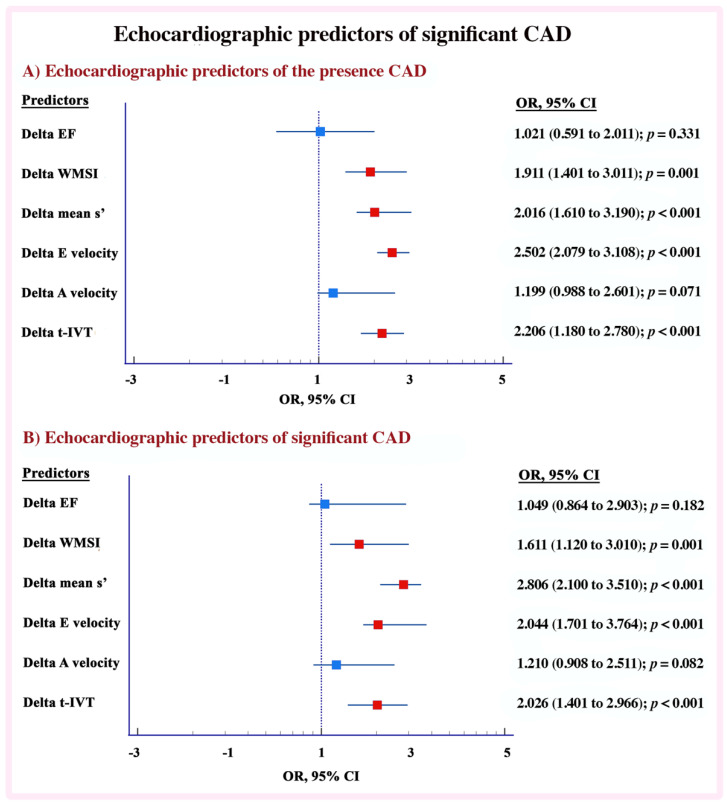
Echocardiographic predictors of significant CAD. (**A**) Echocardiographic predictors of the presence CAD. (**B**) Echocardiographic predictors of significant CAD.

In univariate analysis, Δ EF (*p* = 0.04), Δ WMSI (*p* = 0.001), Δ E/A ratio (*p* = 0.03), Δ A velocity (*p* = 0.01), Δ mean s’ (*p* < 0.001) Δ E wave velocity (*p* = 0.001), Δ A wave velocity (*p*-0.03), Δ t-IVT (*p* < 0.001) and Δ Tei index (*p* = 0.03) predicted the presence of CAD. Δ mean s’, OR 2.016 (1.610 to 3.190; *p* < 0.001), Δ E velocity OR 2.502 (1.179 to 1.108; *p* < 0.001), Δ t-IVT 2.206 (1.180 to 2.780; *p* < 0.001) and Δ mean WMSI OR 1.911 (1.401 to 3.001; *p* = 0.001) independently predicted the presence of CAD on multivariate analysis (Figure 2A and Table S5). On the other hand, Δ EF, Δ WMSI, Δ A wave velocity, Δ mean s’ and Δ E wave velocity, Δ t-IVT, Δ Tei index, Δ MAPSE-s and Δ E/e’ ratio predicted significant CAD in univariate analysis (*p* < 0.05 for all). However, in multivariate analysis, Δ mean s’, Δ E wave velocity, Δ t-IVT and Δ WMSI independently predicted significant CAD (*p* < 0.01) Figure 2B and Table S5). In addition, the accuracy of DSE was higher in significant CAD compared with insignificant CAD (80% vs. 74 %; *p* = 0.03, Table S6, Graphical abstract).

### 3.6. Echocardiographic Predictors of Multivessel and Single Vessel Coronary Disease

Using the ROC analysis, the echocardiographic measurements had highest accuracy in predicting multivessel CAD. A mean s’ < 5.0 cm/s was 87% sensitive, 84% specific with AUC 0.90; *p* < 0.001, and WMSI < 0.7, was 77% sensitive, 78% specific with AUC of 0.86, *p* < 0.001 in predicting multivessel CAD disease. While in predicting of single vessel disease the AUC were 0.88 and 0.77 (respectively, Figure S2). Likewise markers of global LV dyssynchrony predicted multivessel coronary disease (△ t-IVT ≥ 4.5 s/min being 79% sensitive, 78% specific with AUC of 0.84, *p* < 0.001; △ FT ≥ 150 ms 72% sensitive, 68% specific with AUC of 0.72, *p* = 0.01 and △ E wave velocity < 6.0 cm/s, being 72% sensitive, 78% specific with AUC of 0.75, *p* = 0.001 and single vessel disease (△ t-IVT ≥ 4.5 s/min being 77% sensitive, 75% specific with AUC of 0.80, *p* < 0.001; △ FT ≥ 150 ms 70% sensitive, 64% specific with AUC of 0.70, *p* = 0.01 and △ E wave velocity < 6.0 cm/s, being 73% sensitive, 75% specific with AUC of 0.77, *p* < 0.001, Figure S2).

### 3.7. The Effect of LV Cavity Dyssynchrony and Longitudinal Systolic Function in CAD Patients

**Figure 3:**
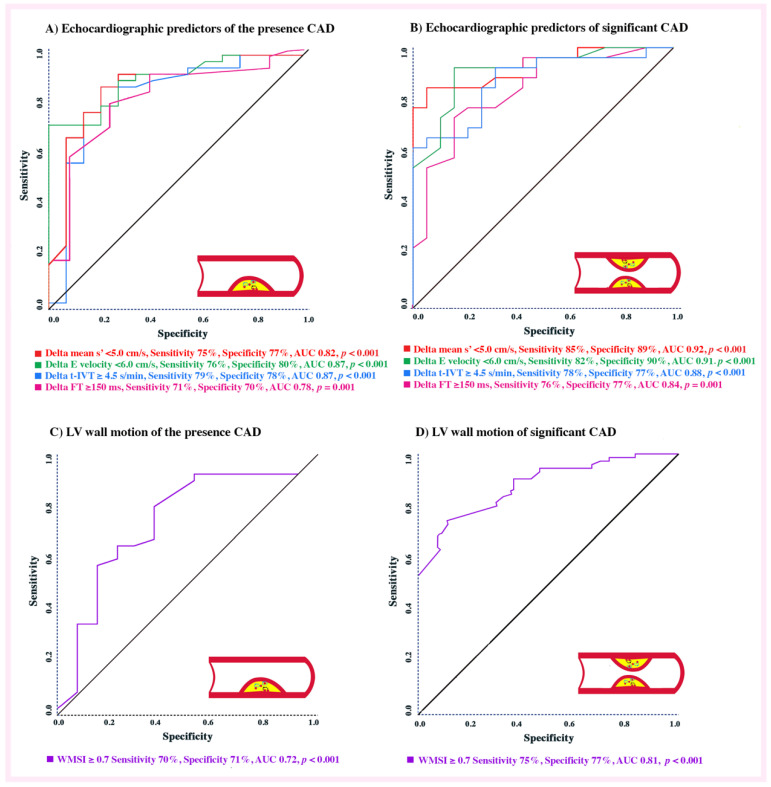
ROC analysis of echocardiographic predictors. (**A**) Echocardiographic predictors of the presence CAD. (**B**) Echocardiographic predictors of significant CAD. (**C**) LV wall motion score index of the presence CAD. (**D**) LV wall motion score index of significant CAD.

Markers of global dyssynchrony predicted CAD with Δ t-IVT ≥ 4.5 s/min being 79% sensitive and 78% specific with AUC of 0.87, *p* < 0.001; Δ FT ≥ 150 ms 71% sensitive and 70% specific with AUC of 0.78, *p* = 0.001 and Δ E wave velocity < 6.0 cm/s, 76% sensitive and 80% specific with AUC of 0.87, *p* < 0.00. Likewise, mean s’ < 5.0 cm/s was 75% sensitive and 77% specific (AUC 0.82; *p* < 0.001), and WMSI < 0.7 was 70% sensitive and 71% specific with AUC of 0.72, *p* < 0.001 in predicting CAD (Figure 3A,C).

Identifying predictors of significant CAD shows the following: mean s’ < 5.0 cm/s was 85% sensitive and 89% specific with AUC of 0.92, *p* < 0.001; WMSI < 0.7 was 75% sensitive and 77% specific with AUC of 0.81, *p* < 0.001; Δ t-IVT ≥ 4.5 s/min was 78% sensitive and 77% specific with AUC of 0.88, *p* < 0.001; Δ FT ≥ 150 ms, 76% sensitive and 77% specific with AUC of 0.84, *p* < 0.001 and Δ E wave velocity < 6.0 cm/s, 82% sensitive and 90% specific with AUC of 0.91, *p* < 0.001 in predicting significant CAD (Figure 3B,D). Thus, the highest accurate predictors of significant CAD (with AUC ≥ 90%) were mean LV s’ < 5.0 cm/s and Δ E wave velocity < 6.0 cm/s.

## 4. Discussion

Findings: This comparative study evaluates the diagnostic role of DSE in predicting the presence of CAD and its severity. Our findings can be summarized as follows: (a) At peak DSE, basal LV dimensions did not differ between groups; (b) the compromised LV longitudinal systolic function and systolic velocities indices (s’) were more pronounced in the CAD compared with no-CAD patients as well as between significant and nonsignificant CAD; (c) in the same group with significant CAD, the LV diastolic function and global markers of dyssynchrony were more distributed; and finally; (d) The most powerful echo predictors (in accuracy order) of presence of CAD were Δ E wave velocity (<6.0 cm/s, AUC = 0.87), t-IVT (≥4.5 s/min; AUC = 0.87), Δ mean s’ (<5.0 cm/s; AUC = 0.82), Δ FT (≥150 ms; AUC = 0.87) and WMSI (≥0.7; AUC = 0.72) and predictors of significant CAD were Δ mean s’ (<5.0 cm/s; AUC = 0.92), Δ E wave velocity (<6.0 cm/s, AUC = 0.91), t-IVT (≥4.5 s/min; AUC = 0.88), Δ FT (≥150 ms; AUC = 0.84) and WMSI (≥0.7; AUC = 0.81).

Data interpretation: Stress echocardiography has been practiced to diagnose myocardial ischemic dysfunction based on segmental wall motion abnormalities in the form of hypo, akinesia or dyskinesia. The use of this 2D modality resulted in an average DSE accuracy of 63–73% in predicting significant coronary artery stenosis [26,27]. The application of tissue Doppler echocardiography during stress echo, although less frequently used, also aims at detecting the development of reduced systolic or early diastolic longitudinal segmental velocities as markers of ischemia. The application of myocardial velocity disturbances during DSE has raised the technique’s accuracy in detecting ischemia to approximately 75–80% [9,28]. The findings of this study introduce a rather different set of echocardiographic markers of ischemia in the form of global attenuated systolic myocardial velocity, compromised early diastolic cavity filling velocity and prolonged total isovolumic time, which reflect the extent of cavity dyssynchrony as a result of induced ischemia. These markers proved to have significantly higher accuracy than the conventional WMSI and segmental myocardial velocities in demonstrating stress induced ischemic dysfunction, secondary to significant coronary artery stenosis. Although these variables are three, they are interrelated. We showed in the 1990s that acute ischemia during coronary balloon inflation results in delayed and compromised myocardial segmental shortening [29]. This delayed segmental function imposes on early diastolic events, prolongs isovolumic relaxation and increases early diastolic cavity tension, hence attenuating its filling velocities. Our current results represent dynamic reproductions of those findings, with the resulting prolonged total isovolumic time, compromised mean LV cavity systolic velocities and also early diastolic filling velocities. These findings support what we have also previously shown: that successful revascularization reverses those abnormalities [30]. Thus, it seems that the very accurate predictor of the development of ischemia during dobutamine stress is based on cavity function disturbances rather than segmental ones. These findings should have valuable relevance in identifying patients with significant coronary stenosis who should benefit from further intervention, rather than predicting single segmental dysfunction at only 76% accuracy, thus leaving a significant percentage of patients undiagnosed. If the latter is the main objective of the functional test, then myocardial perfusion assessment could be a better method.

It is of significant interest that our results confirm other findings in that diastolic dysfunction in the form of reduced delta E wave velocity was the most accurate predictor of the presence of CAD, irrespective of its severity [31,32,33]. This reflects the collective impact of atherosclerosis pathology, including different size branches as well as microcirculation disease, on overall cavity diastolic performance. However, when testing for the most accurate predictor of significant stenosis that could be potentially amenable for revascularization, systolic longitudinal cavity velocities arise as the most accurate predictor. Although in a small number of patients, our findings clearly demonstrate the selective reflection of different components of longitudinal function of the extent of ischemia, the cavity suffers as it increases its oxygen demand during stress.

Clinical implications: While LV wall motion analysis during DSE remains an important noninvasive and radiation-free method for the routine evaluation of patients with suspected coronary artery disease, additional function parameters can increase the accuracy of the investigation in predicting the presence and severity of coronary disease. Early diastolic LV cavity filling velocity attenuation highlights the development of cavity ischemic dysfunction enough to suggest the presence of CAD, irrespective of its severity. Attenuated longitudinal cavity systolic velocity and prolonged total isovolumic time are additional two simple measurements that are 91% accurate in predicting the presence of over >50% coronary branch stenosis. Such measurements, although not built in the routinely used commercial stress echo software, they are routinely acquired in all transthoracic echocardiographic examinations, hence should assist in raising the accuracy of the daily use of such patient-friendly investigation.

Limitations: This study has some limitations. The relatively small number of participants did not allow us to stratify the patients into more subgroups according to the severity and distribution of the coronary disease, as well as prevalence of cardiac risk factors. Details about the myocardial deformation parameters including speckle tracking parameters to compare with were not available. None of our studied patients had conduction disease, so the application of the findings in this subset of patients remains to be tested.

## 5. Conclusions

Compromised LV longitudinal systolic function, lower delta E wave, prolonged total isovolumic time and increased WMSI were the most powerful independent predictors of the presence and significance of CAD. These findings strengthen the role of comprehensive DSE analysis in diagnosing ischemic disturbances secondary to significant CAD.

## Figures and Tables

**Table 1:** Baseline and stress echocardiographic indices in patients with and without CAD.

Variable		Patients with Suspected CAD (*n* = 103)	Patients with CAD − (*n* = 44)	Patients with CAD + (*n* = 59)	*p* Value
LV dimensions					
LVEDD (cm)	Δ (%)	−0.53 ± 0.3 (−11.1)	−0.47 ± 0.3 (−10.1)	−0.54 ± 0.3 (−11.3)	0.11
IVSd (cm)	Δ (%)	0.12 ± 0.1 (11.5)	0.12 ± 0.1 (11.9)	0.11 ± 0.2 (10.5)	0.33
LVPWd (cm)	Δ (%)	0.09 ± 0.1 (10.1)	0.10 ± 0.1 (11.2)	0.08 ± 0.2 (9.0)	0.12
LV systolic function					
LV EF (%)	Δ (%)	6.79 ± 6.1 (11.6)	9.6 ± 4.3 (15.6)	5.7 ± 6.4 (9.9)	0.02
Lateral s’ (cm/s)	Δ (%)	3.8 ± 3.6 (43.2)	5.0 ± 2.6 (56.6)	2.6 ± 1.8 (29.8)	0.001
Septal s’ (cm/s)	Δ (%)	3.2 ± 2.9 (43.0)	4.1 ± 2.6 (54.6)	2.2 ± 2.5 (29.7)	0.004
Posterior s’ (cm/s)	Δ (%)	5.2 ± 4.1 (53.9)	6.7 ±3.9 (64.4)	4.1 ± 3.9 (46.1)	0.003
MAPSEl (cm)	Δ (%)	0.17 ± 0.2 (11.9)	0.20 ± 0.3 (12.9)	0.12 ± 0.2 (9.2)	0.002
MAPSEs (cm)	Δ (%)	0.17 ± 0.2 (13.6)	0.21 ± 0.3 (14.9)	0.12 ± 0.2 (10.9)	0.001
MAPSEp (cm)	Δ (%)	0.24 ± 0.2 (17.1)	0.29 ± 0.3 (18.3)	0.18 ± 0.2 (14.7)	0.001
LV diastolic function					
E wave (cm/s)	Δ (%)	10.3 ± 7.8 (15.5)	15.1 ± 3.1 (22.8)	6.11 ± 4.3 (9.1)	0.001
A wave (cm/s)	Δ (%)	9.1 ± 5.1 (13.7)	12.5 ± 7.4 (19.1)	5.40 ± 5.2 (8.1)	0.02
E/A ratio	Δ (%)	0.02 ± 0.1 (2.0)	0.03 ± 0.5 (2.9)	0.02 ± 0.3 (2.0)	0.11
E/e’ ratio	Δ (%)	−0.34 ± 2.3 (−4.14)	−1.52 ± 1.5 (−17.3)	0.23 ± 2.6 (2.6)	0.01
Lateral e’ (cm/s)	Δ (%)	2.0 ± 2.1 (21.4)	3.7 ± 1.9 (45.1)	0.50 ± 1.6 (5.9)	0.001
Lateral a’ (cm/s)	Δ (%)	2.9 ± 3.4 (28.1)	2.4 ± 2.1 (24.5)	2.6 ± 3.1 (24.3)	0.32
Septal e’ (cm/s)	Δ (%)	2.1 ± 1.7 (31.2)	3.7 ± 1.9 (47.8)	0.5 ± 1.6 (7.7)	0.001
Septal a’ (cm/s)	Δ (%)	3.2 ± 2.1 (34.7)	3.5 ± 2.1 (37.2)	3.0 ± 2.2 (33.3)	0.39
Posterior e’(cm/s)	Δ (%)	3.4 ± 1.7 (37.7)	3.8 ± 1.8 (38.0)	3.0 ± 2.5 (35.9)	0.07
Posterior a’(cm/s)	Δ (%)	3.3 ± 2.9 (30.8)	3.1 ± 2.5 (27.8)	3.3 ± 2.1 (31.4)	0.73
LV global function					
Ejection time (ms)	Δ (%)	−102 ± 11 (34.6)	−105 ± 30 (35.4)	−99 ± 41 (33.1)	0.08
Ejection time(s/ms)	Δ (%)	1.6 ± 1.4 (7.84)	1.0 ± 1.3 (4.76)	2.0 ± 1.4 (10)	0.04
Filling time (ms)	Δ (%)	−157 ± 38 (−34.9)	−148 ± 20 (−32.3)	−169 ± 100 (−37.8)	0.04
Filling time (s/ms)	Δ (%)	1.5 ± 0.9 (4.77)	2.7 ± 1.4 (8.25)	0.4 ± 0.3 (1.32)	0.01
t-IVT (s/min)	Δ (%)	−3.6 ± 1.8 (−40.6)	−4.1 ± 0.9 (−56.2)	−3.1 ± 2.0 (−29.8)	0.03
Tei index (s/min)	Δ (%)	0.13 ± 0.1 (−31.7)	0.11 ± 0.1 (−34.4)	0.16 ± 0.1 (−31.3)	0.01
WMSI (score)	Δ (%)	0.08 ± 0.03 (7.34)	0.02 ± 0.02 (2.0)	0.14 ± 0.1 (11.8)	0.001

Abbreviation: A = atrial diastolic velocity; E = early diastolic filling velocity; e’ = early diastolic myocardial velocity; EDD = end-diastolic dimension; ESD = end-systolic dimension; IVSd = inter-ventricular septum in diastole; l = lateral; LV = left ventricle; MAPSE = mitral annular plane systolic excursion; PWd = parietal wall in diastole; s’ = systolic myocardial velocity; WMSI: wall motion score index; t-IVT: total isovolumic time.

**Table 2:** Baseline and echocardiographic indices among nonsignificant and significant CAD.

Variable		Patients with CAD + (*n* = 59)	Patients with Non Sig CAD (*n* = 29)	Patients with Sig CAD (*n* = 30)	*p* Value
LV dimensions					
LVEDD (cm)	Δ (%)	−0.53 ± 0.3 (−11.1)	−0.51 ± 0.3 (−11.1)	−0.55 ± 0.06 (−11.2)	0.60
IVSd (cm)	Δ (%)	0.11 ± 0.2 (10.5)	0.12 ± 0.04 (11.4)	0.11 ± 0.03 (10.4)	0.85
LVPWd (cm)	Δ (%)	0.08 ± 0.2 (9.0)	0.09 ± 0.3 (9.7)	0.08 ± 0.03 (9.2)	0.11
LV systolic function					
LV EF (%)	Δ (%)	5.7 ± 6.4 (9.9)	6.8 ± 1.3 (13.0)	5.3 ± 1.2 (7.7)	0.04
Lateral s’ (cm/s)	Δ (%)	2.6 ± 1.8 (29.8)	3.6 ± 1.6 (38.7)	1.8 ± 2.2 (21.9)	0.01
Septal s’ (cm/s)	Δ (%)	2.2 ± 2.5 (29.7)	2.8 ± 2.6 (35.9)	1.7 ± 2.3 (24.7)	0.02
Posterior s’(cm/s)	Δ (%)	4.1 ± 3.9 (46.1)	5.6 ± 3.4 (56.7)	2.5 ± 3.7 (31.5)	0.01
MAPSEl (cm)	Δ (%)	0.12 ± 0.2 (9.2)	0.17 ± 0.2 (12.3)	0.09 ± 0.2 (7.4)	0.02
MAPSEs (cm)	Δ (%)	0.12 ± 0.2 (10.9)	0.15 ± 0.03 (13.4)	0.10 ± 0.2 (9.3)	0.01
MAPSEp (cm)	Δ (%)	0.18 ± 0.2 (14.7)	0.23 ± 0.2 (18.4)	0.16 ± 0.3 (13.9)	0.03
LV diastolic function					
E wave (cm/s)	Δ (%)	6.11 ± 4.3 (9.1)	8.5 ± 3.9 (14.9)	6.4 ± 4.1 (10.2)	0.02
A wave (cm/s)	Δ (%)	5.40 ± 5.2 (8.1)	8.5 ± 2.8 (12.3)	4.4 ± 2.0 (6.8)	0.001
E/A ratio	Δ (%)	0.02 ± 0.3 (2.0)	0.03 ± 0.2 (3.1)	0.02 ± 0.1 (1.0)	0.09
E/e’ ratio	Δ (%)	0.23 ± 2.6 (2.6)	0.11 ± 0.5 (1.3)	0.96 ± 0.8 (10.4)	0.001
Lateral e’ (cm/s)	Δ (%)	0.50 ± 1.6 (5.9)	1.1 ± 1.5 (12.7)	0.09 ± 0.6 (1.1)	0.02
Lateral a’ (cm/s)	Δ (%)	2.6 ± 3.1 (24.3)	2.92 ± 2.1 (26.3)	2.25 ± 0.8 (21.6)	0.09
Septal e’ (cm/s)	Δ (%)	0.5 ± 1.6 (7.7)	0.9 ± 1.8 (13.8)	0.06 ± 1.1 (0.9)	0.01
Septal a’ (cm/s)	Δ (%)	3.0 ± 2.2 (33.3)	3.33 ± 2.4 (35.9)	2.65 ± 0.5 (30.3)	0.57
Posterior e’ (cm/s)	Δ (%)	3.0 ± 2.5 (35.9)	4.3 ± 2.3 (51.8)	2.39 ± 2.5 (28.5)	0.03
Posterior a’ (cm/s)	Δ (%)	3.3 ± 2.1 (31.4)	3.30 ± 2.6 (26.2)	3.38 ± 2.6 (36.2)	0.47
LV global function					
Ejection time (ms)	Δ (%)	−100 ± 41 (−33.3)	−79 ± 40 (−27.8)	−114 ± 36 (−36.8)	0.01
Ejection time (ms) (s/min)	Δ (%)	−100 ± 41 (−33.4)	−79 ± 40 (−27.8)	−114 ± 36 (−36.8)	0.001
Filling time (ms)	Δ (%)	−169 ± 100 (−37.8)	−125 ± 80 (−30.7)	−199 ± 90 (−41.9)	0.001
Filling time (s/min)	Δ (%)	0.4 ± 0.3 (4.65)	1.8 ± 1.2 (5.9)	0.9 ± 0.4 (2.9)	0.08
t-IVT (s/min)	Δ (%)	−3.1 ± 2.0 (−29.8)	−3.6 ± 2.3 (−37.1)	−2.7 ± 1.9 (−24.1)	0.01
Tei index (s/min)	Δ (%)	−0.16 ± 0.1 (−31.4)	−0.16 ± 0.1 (−32.6)	−0.12 ± 0.1 (−23.5)	0.01
WMSI score	Δ (%)	0.14 ± 0.1 (11.8)	0.09 ± 0.01 (8.25)	0.19 ± 0.02 (14.9)	0.001

Abbreviation: A = atrial diastolic velocity; E = early diastolic filling velocity; e’ = early diastolic myocardial velocity; EDD = end-diastolic dimension; ESD = end-systolic dimension; IVSd = inter-ventricular septum in diastole; l = lateral; LV = left ventricle; MAPSE = mitral annular plane systolic excursion; PWd = parietal wall in diastole; s’ = systolic myocardial velocity; WMSI: wall motion score index; t-IVT: total isovolumic time.

## Data Availability

The data presented in this study are available on request from the corresponding author.

## Supplementary Materials

The following supporting information can be downloaded at: https://www.mdpi.com/article/10.3390/jcm11206185/s1, Figure S1: Flow chart of patients; Figure S2: Echocardiographic predictors of multivessel and single vessel disease; Table S1: Demographic, clinical and stress end point among patients; Table S2: Baseline and stress echocardiographic indices in patients with and without CAD; Table S3: Baseline and echocardiographic indices among non significant and significant CAD; Table S4: Clinical predictors of coronary artery disease; Table S5: Echocardiographic predictors of coronary artery disease; Table S6: The accuracy of dobutamine stress echocardiography.
