# Supplementary material for: Left Ventricular Myocardial and Cavity Velocity Disturbances Are Powerful Predictors of Significant Coronary Artery Stenosis
Source: J Clin Med. 2022 Oct 20;11(20):6185. doi: 10.3390/jcm11206185 (PMC9605655; doi:10.3390/jcm11206185)
Supplement: Supplementary file 1 [file jcm-11-06185-s001.zip › jcm-1953804-supplementary.pdf]

Supplemental data

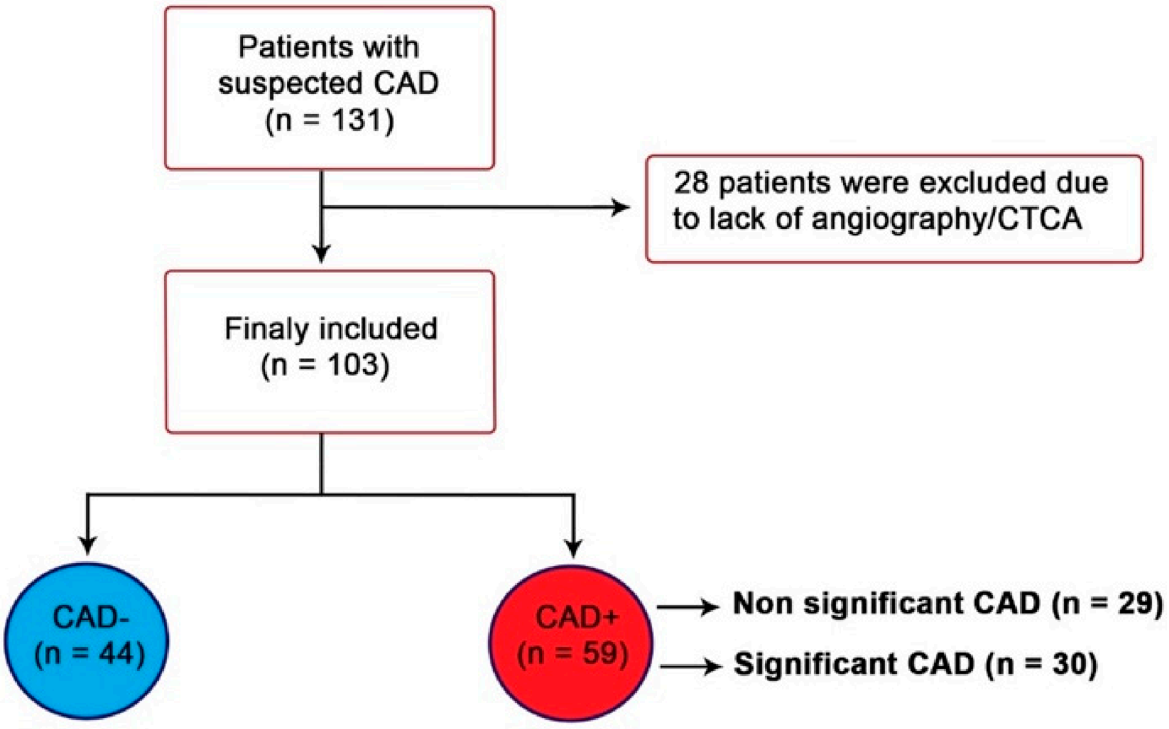

Figure S1. Flow chart of patients

Table S1. Demographic, clinical and stress end point among patients.

Variable	Patients with suspected CAD (*n* = 103)	Patients with CAD - (n = 44)	Patients with CAD + (*n* = 59)	*P* value
Demographic and clinical indices				
Age (years)	60.4 ± 11	56 ± 11	63 ± 11	0.01
Female gender (n, %)	47 (45.6)	29 (63)	18 (30.5)	0.001
SBP (mmHg)	132 ± 23	131 ± 21	135 ± 23	0.82
DBP (mmHg)	72 ± 11	71 ± 12	78 ± 11	0.13
HR	68 ± 10	71 ± 7.0	67 ± 11	0.17
Sinus rhythm (n, %)	103 (100)	46 (100)	59 (100)	0.93
Chest pain (n, %)	91 (88.3)	43 (93.4)	50 (86.2)	0.11
Typical (n, %)	39 (37.8)	30 (69.7)	30 (50.8)	0.01
Atypical (n, %)	52 (50.4)	13 (37.2)	20 (40)	0.21
Cardiovascular risk factors				
Smoker (n, %)	51 (49.5)	23 (50)	28 (47.5)	0.89
AH (n, %)	65 (63.1)	23 (50)	43 (72.8)	< 0.001
DM (n, %)	24 (23.3)	6 (13.1)	18 (30.5)	0.001
Dyslipidemia	69 (67.1)	23 (50)	47 (79.7)	< 0.001
Family history for CHD (n, %)	52 (50.4)	10 (21.7)	26 (44.1)	0.001
Number of cardiovascular risk factors				
0–1	29 (28.1)	22 (47.8)	7 (11.8)	< 0.001
2–3	66 (64.1)	22 (47.8)	39 (66.1)	0.001
> 3	15 (14.5)	3 (6.5)	12 (20.3)	0.001

Stress end point				
Dobutamine dose (ug/kg per min)	32.1 ± 7.9	32.5 ± 7.3	31.9 ± 8.2	0.81
Breathlessness (n, %)	12 (11.9)	0 (0)	9 (15.7)	<0.001
Chest pain (n, %)	26 (25.2)	5 (11.3)	21 (35.5)	0.001
Arrhythmia (n, %)	16 (15.5)	4 (9.09)	12 (20.3)	0.01
Hypotensive (n, %)	3 (2.9)	0 (0)	3 (5.08)	0.03
T, ST-segment change (n, %)	16 (16.2)	3 (6.81)	13 (22.0)	0.01
DSE positive (n, %)	50 (48.5)	7 (15.9)	43 (71.9)	<0.001

AH: arterial hypertension; CHD; coronary heart disease; DM: diabetes mellitus; SBP: systolic blood pressure; DBP: Diastolic blood pressure

Table S2. Baseline and stress echocardiographic indices in patients with and without CAD

**Variable**		**Patients with suspected CAD (*n* = 105)**	**Patients with CAD - (*n* = 46)**	**Patients with CAD + (*n* = 59)**	***P* value**
		LV dimensions			
LVEDD (cm)	Rest	4.78 ± 0.5	4.78 ± 0.4	4.77 ± 0.6	0.72
	Stress	4.26 ± 0.6	4.31 ± 0.5	4.24 ± 0.6	0.22
	Delta	-0.53 ± 0.3	-0.47 ± 0.3	-0.54 ± 0.3	0.11
	P value	<0.001	0.001	<0.001	
IVSd (cm)	Rest	1.04 ± 0.1	1.01 ± 0.2	1.05 ± 0.2	0.43
	Stress	1.16 ± 0.2	1.13 ± 0.2	1.17 ± 0.3	0.67
	Delta	0.12 ± 0.1	0.12 ± 0.1	0.11 ± 0.2	0.33
	P value	0.001	0.001	0.001	
LVPWd (cm)	Rest	0.89 ± 0.1	0.89 ± 0.2	0.89 ± 0.2	0.77
	Stress	0.99 ± 0.2	1.0 ± 0.2	0.98 ± 0.2	0.12
	Delta	0.09 ± 0.1	0.10 ± 0.1	0.08 ± 0.2	0.12
	P value	0.02	0.01	0.02	
		LV systolic function			
LV EF (%)	Rest	58.3 ± 6.5	60.4 ± 2.2	58.2 ± 7.2	0.11
	Stress	65.1 ± 10	70.0 ± 4.6	63.9 ± 11	0.03
	Delta	6.79 ± 6.1	9.6 ± 4.3	5.7 ± 6.4	0.02
	P value	<0.001	<0.001	0.001	
Laterals' (cm/s)	Rest	8.77 ± 2.3	8.83 ± 2.7	8.72 ± 1.8	0.33
	Stress	12.6 ± 3.5	13.9 ± 3.6	11.3 ± 3.2	0.01
	Delta	3.8 ± 3.6	5.0 ± 2.6	2.6 ± 1.8	0.001
	P value	<0.001	<0.001	<0.001	
Septal s' (cm/s)	Rest	7.44 ± 1.6	7.51 ± 1.6	7.38 ± 1.7	0.12
	Stress	10.6 ± 3.3	11.6 ± 2.9	9.6 ± 3.1	0.02
	Delta	3.2 ± 2.9	4.1 ± 2.6	2.2 ± 2.5	0.004
	P value	< 0.001	< 0.001	< 0.001	
Posterior s' (cm/s)	Rest	9.64 ± 2.3	10.4 ± 2.2	8.89 ± 2.1	0.11
	Stress	14.9 ± 4.9	17.1 ± 4.4	12.9 ± 4.5	0.002
	Delta	5.2 ± 4.1	6.7 ±3.9	4.1 ± 3.9	0.003
	P value	< 0.001	< 0.001	< 0.001	
MAPSEl (cm)	Rest	1.43 ± 0.3	1.56 ± 0.3	1.31 ± 0.2	0.04
	Stress	1.60 ± 0.3	1.78 ± 0.3	1.42 ± 0.3	0.01
	Delta	0.17 ± 0.2	0.20 ±0.3	0.12 ± 0.2	0.002
	P value	< 0.001	0.02	0.01	
MAPSEs (cm)	Rest	1.25 ± 0.2	1.41 ± 0.2	1.10 ± 0.2	0.01
	Stress	1.42 ± 0.3	1.62 ± 0.3	1.22 ± 0.3	0.01
	Delta	0.17 ± 0.2	0.21 ± 0.3	0.12 ± 0.2	0.001

	P value	< 0.001	< 0.001	< 0.001	
MAPSEp (cm)	Rest	1.40 ± 0.3	1.58 ± 0.4	1.22 ± 0.4	0.04
	Stress	1.64 ± 0.3	1.88 ± 0.3	1.40 ± 0.3	0.02
	Delta	0.24 ± 0.2	0.29 ± 0.3	0.18 ± 0.2	0.001
	P value	<0.001	0.001	0.02	
			LV diastolic function		
E wave (cm/s)	Rest	66.5 ± 12	66.1 ± 11	67.2 ± 12	0.33
	Stress	76.7 ± 13	81.2 ± 13	73.3 ± 13	0.01
	Delta	10.3 ± 7.8	15.1 ± 3.1	6.11 ± 4.3	0.001
	P value	0.03	<0.001	0.03	
A wave (cm/s)	Rest	66.3 ± 14	65.6 ± 10	67.3 ± 13	0.14
	Stress	75.4 ± 11	78.1 ± 7.2	72.7 ± 14	0.09
	Delta	9.1 ± 5.1	12.5 ± 7.4	5.40 ± 5.2	0.001
	P value	< 0.001	< 0.001	0.01	
E/A ratio	Rest	1.0 ± 0.2	1.01 ± 0.4	0.99 ± 0.3	0.11
	Stress	1.02 ± 0.2	1.04 ± 0.5	1.01 ± 0.3	0.02
	Delta	0.02 ±0.1	0.03 ± 0.5	0.02 ± 0.3	0.01
	P value	0.04	0.04	0.01	
E/e′ ratio	Rest	8.20 ± 2.6	8.87 ± 2.4	8.94 ± 2.6	0.44
	Stress	7.85 ± 2.8	7.34 ± 1.5	9.17 ± 3.1	0.04
	Delta	−0.34 ± 2.3	−1.52 ± 1.5	0.23 ± 2.6	0.01
	P value	0.01	0.001	0.04	
Lateral e′ (cm/s)	Rest	8.33 ± 2.9	8.20 ± 3.3	8.52 ± 2.6	0.21
	Stress	10.3 ± 3.3	11.9 ± 3.4	9.02 ± 2.9	0.72
	Delta	2.0 ± 2.1	3.7 ± 1.9	0.50 ± 1.6	0.64
	P value	0.005	0.002	0.11	
Lateral a′ (cm/s)	Rest	10.3 ± 2.9	9.79 ± 2.7	10.7 ± 2.4	0.44
	Stress	13.3 ± 3.5	13.2 ± 3.5	13.3 ± 3.4	0.41
	Delta	2.9. ± 3.4	2.4 ± 2.1	2.6 ± 3.1	0.32
	P value	0.01	0.02	0.01	
Septal e′ (cm/s)	Rest	6.73 ± 1.7	6.90 ± 1.5	6.53 ± 1.8	0.35
	Stress	8.80 ± 2.2	10.2 ± 2.1	7.02 ± 2.1	0.28
	Delta	2.1 ± 1.7	3.3 ±1.8	0.49 ± 1.4	0.09
	P value	0.006	0.01	0.02	
Septal a′ (cm/s)	Rest	9.20 ± 1.9	9.4 ± 1.8	9.0 ± 2.1	0.81
	Stress	12.4 ± 2.9	12.9 ± 2.9	12.0 ± 2.7	0.77
	Delta	3.2 ± 2.1	3.5 ± 2.1	3.0 ± 2.2	0.39
	P value	<0.001	<0.001	0.001	
Posterior e′ (cm/s)	Rest	9.01 ± 3.1	10.0 ± 2.9	8.35 ± 2.2	0.04
	Stress	12.5 ± 4.2	13.8 ± 3.2	11.4 ± 4.3	0.04
	Delta	3.5 ± 1.7	3.8 ± 1.8	3.0 ± 2.5	0.07
	P value	0.008	0.006	0.002	
Posterior a′ (cm/s)	Rest	10.7 ± 2.7	11.2 ± 1.7	10.5 ± 3.2	0.28
	Stress	14.0 ± 2.4	14.3 ± 2.4	13.8 ± 3.2	0.33
	Delta	3.3 ± 2.9	3.1 ± 2.5	3.3 ± 2.1	0.73
	P value	< 0.001	0.001	0.001	
			LV global function		
Ejection time (ms)	Rest	298 ± 37	296 ± 36	299 ± 38	0.22
	Stress	195 ± 26	191 ± 32	199 ± 22	0.31
	Delta	102 ± 11	105 ± 30	100 ± 41	0.08
	P value	< 0.001	< 0.001	< 0.001	
Ejection time	Rest	20.4 ± 3.7	21 ± 2.4	20 ± 4.1	0.11

(s/ms)	Stress	22.0 ± 2.9	22 ± 3.8	22 ± 2.4	0.10
	Delta	1.6 ± 1.4	1.0 ± 1.3	2.0 ± 1.4	0.04
	P value	0.04	0.04	0.02	
Filling time (ms)	Rest	450 ± 106	458 ± 86	447 ± 113	0.09
	Stress	293 ± 59	309 ± 43	277 ± 65	0.03
	Delta	157 ±38	148 ±20	169 ±100	0.04
	P value	< 0.001	< 0.001	< 0.001	
Filling time (s/ms)	Rest	31.4 ± 2.2	32.7 ± 5.8	30.1 ± 2.2	0.04
	Stress	32.9 ± 6.4	35.4 ± 5.1	30.5 ± 7.2	0.01
	Delta	1.5 ±0.9	2.7 ±1.4	0.4 ± 0.3	0.01
	P value	0.01	0.001	0.04	
t-IVT (s/min)	Rest	8.87 ± 3.4	7.29 ± 1.3	10.4. ± 3.2	0.01
	Stress	5.21 ± 3.0	3.20 ± 1.1	7.30 ± 3.1	0.01
	Delta	−3.6 ± 1.8	−4.1 ±0.9	−3.1 ±2.0	0.03
	P value	0.001	0.001	0.01	
Tei index (s/min)	Rest	0.41 ± 0.2	0.32 ± 0.2	0.51 ± 0.2	0.02
	Stress	0.28 ± 0.1	0.21 ± 0.1	0.35 ± 0.1	0.02
	Delta	0.13 ± 0.1	0.11 ± 0.1	0.16 ±0.1	0.04
	P value	0.01	0.02	0.001	
WMSI (score)	Rest	1.09 ± 0.18	1.0 ± 0.01	1.18 ± 0.2	0.01
	Stress	1.16 ± 0.21	1.02 ± 0.02	1.32 ± 0.3	0.001
	Delta	0.08 ±0.03	0.02 ± 0.02	0.14 ± 0.1	0.0011
	P value	0.001	0.26	0.01	0.04

Abbreviation: A = atrial diastolic velocity; E = early diastolic filling velocity; e′ = early diastolic myocardial velocity; EDD = end-diastolic dimension; ESD = end-systolic dimension; IVSd = inter-ventricular septum in diastole; l = lateral; LV = left ventricle; MAPSE = mitral annular plane systolic excursion; PWd = parietal wall in diastole; s′ = systolic myocardial velocity; WMSI: wall motion score index; t-IVT: total isovolemic time.

Table S3. Baseline and echocardiographic indices among non significant and significant CAD.

**Variable**		**Patients with CAD + (*n* = 59)**	**Patients with non sig CAD (*n* = 29)**	**Patients with sig CAD (*n* = 30)**	***P* value**
			LV dimensions		
LVEDD (cm)	Rest	4.78 ± 0.6	4.57 ± 0.4	4.91 ± 0.6	0.66
	Stress	4.24 ± 0.6	4.06 ± 0.5	4.36 ± 0.6	0.22
	Delta	−0.54 ± 0.3	−0.51 ± 0.3	−0.55 ± 0.06	0.60
	P value	< 0.001	< 0.001	< 0.001	
IVSd (cm)	Rest	1.05 ± 0.2	1.05 ± 0.2	1.05 ± 0.2	0.91
	Stress	1.17 ± 0.3	1.17 ± 0.2	1.16 ± 0.3	0.80
	Delta	0.11 ± 0.2	0.12 ± 0.04	0.11 ± 0.03	0.85
	P value	0.02	0.01	0.02	
LVPWd (cm)	Rest	0.89 ± 0.2	0.92 ± 0.2	0.87 ± 0.1	0.09
	Stress	0.98 ± 0.2	1.01 ± 0.2	0.95 ± 0.2	0.08
	Delta	0.08 ± 0.2	0.09 ± 0.3	0.08 ±0.03	0.11
	P value	0.04	0.03	0.04	
			LV systolic function		
LV EF (%)	Rest	57.2 ± 7.2	59.7 ± 3.0	55.4 ± 8.6	0.09
	Stress	62.9 ± 11	67.5 ± 6.8	59.7 ± 13	0.04
	Delta	5.7 ± 6.4	7.8 ± 1.3	4.3 ± 1.2	0.04
	P value	0.01	0.001	0.01	
Lateral s′ (cm/s)	Rest	8.72 ± 1.8	9.30 ± 1.9	8.20 ± 1.7	0.06

	Stress	11.3 ± 3.2	12.9 ± 3.3	10.0 ± 2.1	0.03
	Delta	2.6 ± 1.8	3.6 ± 1.6	1.8 ± 2.2	0.01
	P **value**	0.001	<0.001	0.01	
Septal s′ (cm/s)	Rest	7.38 ± 1.7	7.80 ± 1.8	6.86 ±1.5	0.07
	Stress	9.6 ± 3.1	10.6 ± 3.3	8.61 ± 2.1	0.03
	Delta	2.2 ± 2.5	2.8 ± 2.6	1.7 ± 2.3	0.02
	P value	< 0.001	< 0.001	< 0.001	
Posterior s′ (cm/s)	Rest	8.89 ± 2.1	9.87 ± 1.3	7.94 ±2.4	0.06
	Stress	12.9 ± 4.5	15.5 ± 3.3	10.4 ± 4.8	0.01
	Delta	4.1 ± 3.9	5.6 ± 3.4	2.5 ± 3.7	0.01
	P value	0.001	< 0.001	0.01	
MAPSEl (cm)	Rest	1.31 ± 0.2	1.38 ± 0.1	1.22 ± 0.2	0.03
	Stress	1.42 ± 0.3	1.55 ± 0.2	1.31 ± 0.2	0.02
	Delta	0.12 ± 0.2	0.17 ± 0.2	0.09 ± 0.2	0.02
	P value	0.03	0.01	0.03	
MAPSEs (cm)	Rest	1.10 ± 0.2	1.14 ± 0.2	1.06 ± 0.2	0.04
	Stress	1.22 ± 0.3	1.29 ± 0.3	1.16 ± 0.3	0.03
	Delta	0.12 ± 0.2	0.15 ±0.03	0.10 ± 0.2	0.01
	P value	0.001	0.001	0.02	
MAPSEp (cm)	Rest	1.22 ± 0.4	1.25 ± 0.2	1.15 ± 0.5	0.03
	Stress	1.40 ± 0.3	1.48 ± 0.3	1.32 ± 0.4	0.03
	Delta	0.18 ± 0.2	0.23 ± 0.2	0.16 ± 0.3	0.03
	P value	0.02	0.001	0.02	
		LV diastolic function			
E wave (cm/s)	Rest	67.2 ± 12	63.7 ± 12	68.8 ± 13	0.10
	Stress	73.3 ± 13	72.2 ± 10	75.2 ± 14	0.11
	Delta	6.11 ± 4.3	8.5 ± 3.9	6.4 ± 4.1	0.02
	P value	0.03	<0.001	0.01	
A wave (cm/s)	Rest	67.3 ± 13	68.8 ± 23	64.2 ± 15	0.21
	Stress	72.7 ± 14	77.3 ± 26	68.6 ± 15	0.01
	Delta	4.70 ± 5.2	8.5 ± 2.8	4.4 ± 2.0	0.001
	P value	0.03	0.01	0.03	
E/A ratio	Rest	0.99 ± 0.3	0.95 ± 0.4	1.04 ± 0.3	0.22
	Stress	1.01 ± 0.3	0.97 ± 0.5	1.05 ± 0.3	0.34
	Delta	0.02 ± 0.3	0.03 ± 0.1	0.01 ± 0.1	0.09
	P value	0.13	0.11	0.16	
E/e′ ratio	Rest	8.94 ± 2.6	8.40 ± 2.3	9.24 ± 2.2	0.04
	Stress	9.17 ± 3.1	8.51 ± 3.6	10.2 ± 2.6	0.01
	Delta	0.23 ± 2.6	0.11 ± 0.5	0.96 ± 0.8	0.01
	P value	0.04	0.41	0.03	
Lateral e′ (cm/s)	Rest	8.52 ± 2.6	8.59 ± 2.5	8.33 ± 2.3	0.20
	Stress	9.02 ± 2.9	9.61 ± 3.4	8.42 ± 2.6	0.41
	Delta	0.50 ± 1.6	1.1 ± 1.5	0.09 ± 0.6	0.02
	P value	0.04	0.04	0.42	
Lateral a′ (cm/s)	Rest	10.7 ± 2.4	11.1 ± 2.9	10.4 ± 1.8	0.22
	Stress	13.3 ± 3.4	14.0 ± 3.4	12.6 ± 3.1	0.04
	Delta	2.6 ± 3.1	2.9 ± 2.1	2.2 ± 0.8	0.09
	P value	0.01	0.03	0.04	
Septal e′ (cm/s)	Rest	6.53 ± 1.8	6.49 ± 1.8	6.55 ± 1.5	0.33
	Stress	7.02 ± 2.1	7.40 ±2.3	6.61 ± 1.3	0.19
	Delta	0.49 ± 1.4	0.9 ± 1.8	0.06 ± 1.1	0.01
	P value	0.04	0.01	0.22	

Septal a′ (cm/s)	Rest	9.0 ± 2.1	9.26 ± 2.3	8.73 ± 1.9	0.11
	Stress	12.0 ± 2.7	12.6 ±2.6	11.4 ± 2.6	0.20
	Delta	3.0 ± 2.2	3.33 ± 2.4	2.65 ± 0.5	0.57
	***P* value**	0.01	0.01	0.02	
Posterior e′ (cm/s)	Rest	8.35 ± 2.2	8.30 ± 2.5	8.38 ± 3.2	0.33
	Stress	11.4 ± 4.3	12.6 ±4.8	10.8 ±4.1	0.04
	Delta	3.0 ± 2.5	4.3 ± 2.3	2.3 ± 2.5	0.03
	***P* value**	0.002	0.001	0.01	
Posterior a (cm/s)	Rest	10.5 ± 3.2	12.6 ± 3.3	9.33 ± 2.8	0.04
	Stress	13.8 ± 3.2	15.9 ± 3.3	12.7 ±1.9	0.04
	Delta	3.3 ± 2.1	3.3 ± 2.6	3.4 ± 2.6	0.47
	***P* value**	0.02	0.03	0.02	
		LV global function			
Ejection time (ms)	Rest	299 ± 38	284 ± 33	310 ± 38	0.02
	Stress	199 ± 22	205 ± 24	196 ± 20	0.10
	Delta	−100 ± 41	−79 ± 40	−114 ± 36	0.001
	P value	< 0.001	< 0.001	< 0.001	
Ejection time (s/min)	Rest	20 ± 4.1	20.4 ± 3.6	19.7 ± 3.5	0.11
	Stress	22 ± 2.4	23.4 ± 2.9	22.1 ± 2.1	0.02
	Delta	2.0 ± 1.7	3.4 ± 2.5	2.4 ± 1.9	0.01
	P value	0.01	0.001	0.01	
Filling time (ms)	Rest	447 ± 113	407 ± 123	474 ± 100	0.01
	Stress	277 ± 65	282 ± 71	275 ± 57	0.20
	Delta	−169 ±100	−125±80	−199±90	0.001
	P value	< 0.001	< 0.001	< 0.002	
Filling time (s/min)	Rest	30.1 ± 2.2	30.1 ± 3.6	30.2 ± 3.1	0.51
	Stress	30.5 ± 7.2	31.9 ± 3.4	31.1 ± 5.7	0.44
	Delta	0.4 ± 0.3	1.8 ± 1.2	0.9 ± 0.4	0.08
	P value	0.04	0.01	0.03	
t-IVT (s/min)	Rest	10.4. ± 3.2	9.7 ± 3.7	11.2 ± 3.5	0.10
	Stress	7.30 ± 3.1	6.13 ± 2.8	8.42 ± 3.7	0.04
	Delta	−3.1 ±2.0	−3.6 ± 2.3	−2.7 ±1.9	0.01
	P value	0.01	0.009	0.03	
Tei index (s/min)	Rest	0.51 ± 0.2	0.49 ± 0.1	0.51 ± 0.2	0.01
	Stress	0.35 ± 0.1	0.33 ± 0.1	0.38 ± 0.3	0.04
	Delta	−0.16 ±0.1	−0.16 ± 0.1	−0.12 ± 0.1	0.001
	P value	0.001	0.001	0.01	
WMSI (score)	Rest	1.18 ± 0.2	1.09 ± 0.06	1.27 ± 0.2	0.01
	Stress	1.32 ± 0.3	1.18 ± 0.17	1.46 ± 0.3	0.01
	Delta	0.14 ± 0.1	0.09 ±0.01	0.19 ± 0.02	0.001
	P value	0.01	0.02	0.001	

Abbreviation: A = atrial diastolic velocity; E = early diastolic filling velocity; e′ = early diastolic myocardial velocity; EDD = end-diastolic dimension; ESD = end-systolic dimension; IVSd = inter-ventricular septum in diastole; l = lateral; LV = left ventricle; MAPSE = mitral annular plane systolic excursion; PWd = parietal wall in diastole; s′ = systolic myocardial velocity; WMSI: wall motion score index; t-IVT: total isovolemic time.

Table S4. Clinical predictors of coronary artery disease

**Variable**	**Univariate predictors OR (95% CI)**	***P* value**	**Multivariate predictors OR (95% CI)**	***P* value**
	**In predicting CAD +**			
Age	1.159 (1.029 to 1.805)	0.002	3.044 (2.189 to 5.010)	0.01

Female gender	3.025 (1.756 to 8.236)	0.001	2.162 (1.589 to 2.124)	0.01
Diabetes	2.643 (0.981 to 5.021)	0.03	1.185 (0.491 to 2.001)	0.11
AH	1.881 (0.858 to 2.008)	0.06		
Dyslipidemia	2.190 (1.798 to 4.103)	0.001	1.224 (0.890 to 3.132)	0.06
Smoker	2.080 (0.800 to 5.010)	0.22		
Family history for CAD	0.871 (0.331 to 1.106)	0.31		
Number of risk factors	3.681 (2.309 to 4.105)	< 0.001	3.701 (2.410 to 4.511)	< 0.001
**In predicting significant CAD**				
Age	1.178 (1.045 to 2.996)	0.02	1.101 (0.881 to 3.696)	0.10
Female gender	2.143 (0.045 to 4.196)	0.001	2.812 (1.601 to 4.006)	0.001
Diabetes	2.122 (1.501 to 3.037)	0.03	1.101 (0.901 to 2.996)	0.22
AH	1.703 (0.919 to 3.019)	0.09		
Dyslipidemia	2.351 (1.502 to 4.013)	0.01	1.301 (0.820 to 3.033)	0.18
Smoker	1.020 (0.898 to 2.011)	0.22		
Family history for CAD	0.912 (0.288 to 1.309)	0.41		
Number of risk factors	2.009 (1.305 to 3.901)	< 0.001	2.311 (1.610 to 4.122)	< 0.001

AH: arterial hypertension; CAD: Coronary artery disease; DM: diabetes mellitus.

Table S5. Echocardiographic predictors of coronary artery disease

Variable	Univariate predictors OR (95% CI)	P value	Multivariate predictors OR (95% CI)	P value
**In predicting CAD +**				
Delta LVEDD	1.570 (0.329 to 4.205)	0.57		
Delta LVPWd	1.896 (0.856 to 6.012)	0.33		
Delta IVSd	0.920 (0.180 to 3.111)	0.47		
Delta EF	1.210 (1.058 to 2.107)	0.04	1.011 (0.701 to 2.010)	0.331
Delta WMSI	2.190 (1.798 to 4.103)	0.001	1.911 (1.401 to 2.710)	0.001
Delta MAPSEl	1.779 (0.150 to 9.810)	0.64		
Delta MAPSEs	2.171 (1.331 to 4.106)	0.02		
Delta MAPSEp	1.171 (1.012 to 2.816)	0.002		
Delta mean s′	2.190 (1.540 to 3.651)	< 0.001	2.016 (1.610 to 3.190)	< 0.001
Delta lateral e′	1.037 (0.881 to 1.676)	0.11		
Delta septal e′	1.332 (1.009 to 1.900)	0.04		
Delta E/e′ ratio	0.990 (0.540 to 1.651)	0.12		
Delta lateral a′	1.123 (0.900 to 2.344)	0.11		
Delta septal a′	1.075 (0.900 to 2.331)	0.22		
Delta E velocity	1.999 (1.2020 to 3.101)	0.001	1.502 (2.079 to 3.108)	< 0.001
Delta A velocity	1.159 (1.090 to 1.897)	0.04	1.199 (0.988 to 2.601)	0.071
Delta E/A ratio	1.599 (1.101 to 2.797)	0.03		
Delta FT	0.977 (0.700 to 0.992)	0.01		
Delta ET	0.901 (0.686 to 1.014)	0.10		
Delta t-IVT	2.102 (1.788 to 3.809)	< 0.001	2.206 (1.180 to 2.780)	< 0.001
Delta Tei index	1.117 (1.010 to 2.800)	0.03		
**In predicting significant CAD**				
Delta LVEDD	2.883 (0.545 to 11.99)	0.27		
Delta LVPWd	1.389 (0.145 to 9.806)	0.66		
Delta IVSd	0.166 (0.077 to 2.011)	0.31		
Delta EF	1.059 (0.064 to 2.166)	0.03	1.049 (0.864 to 2.903)	0.182
Delta WMSI	1.251 (1.068 to 2.313)	0.01	1.611 (1.120 to 3.010)	0.001
Delta MAPSEl	2.620 (1.898 to 4.011)	0.03		
Delta MAPSEs	1.301 (1.118 to 2.111)	0.02		
Delta MAPSEp	1.020 (0.708 to 1.911)	0.10		

Delta mean s′	2.481 (1.682 to 3.012)	0.001	2.806 (2.100 to 3.510	< 0.001
Delta lateral e′	0.996 (0.671 to 1.412)	0.22		
Delta septal e′	4.306 (2.171 to 8.002)	0.02		
Delta E/e′ ratio	1.212 (1.1088 to 2.893)	0.04		
Delta lateral a′	1.063 (0.692 to 1.990)	0.09		
Delta septal a′	1.551 (0.878 to 2.100)	0.23		
Delta posterior a′	1.055 (0.992 to 1.981)	0.39		
Delta E velocity	1.232 (1.084 to 1.701)	0.001	2.044 (1.701 to 3.764)	< 0.001
Delta A velocity	1.048 (0.989 to 1.511)	0.08	1.210 (0.908 to 2.511)	0.082
Delta E/A ratio	1.808 (1.209 to 2.315)	0.01		
Delta FT	1.218 (1.089 to 1.925)	0.01		
Delta ET	1.105 (1.042 to 2.011)	0.01		
Delta t-IVT	1.409 (1.108 to 2.231)	0.01	2.026 (1.401 to 2.966)	< 0.001
Delta Tei index	1.212 (1.035 to 2.001)	0.01		

Abbreviation: A = atrial diastolic velocity; E = early diastolic filling velocity; e′ = early diastolic myocardial velocity; EDD = end-diastolic dimension; ESD = end-systolic dimension; IVSd = inter-ventricular septum in diastole; l = lateral; LV = left ventricle; MAPSE = mitral annular plane systolic excursion; PWd = parietal wall in diastole; s′ = systolic myocardial velocity; WMSI: wall motion score index; t-IVT: total isovolemic time.

Table S6. The accuracy of dobutamine stress echocardiography.

DSE	Presence of CAD	Significant CAD
Sensitivity (%)	70.1 (58 to 82)	83.4 (66 to 94)
Specificity (%)	81.2 (70 to 93)	76.1 (56 to 89)
PPV (%)	84.0 (74 to 932	78.1 (64 to 87)
NPV (%)	66.8 (58 to 76)	81.5 (65 to 89)
Accuracy	74.2 (68 to 84)	80.0 (67 to 89)

Abbreviation: CAD=coronary artery disease; PPV=positive predictive value; NPV=negative predictive

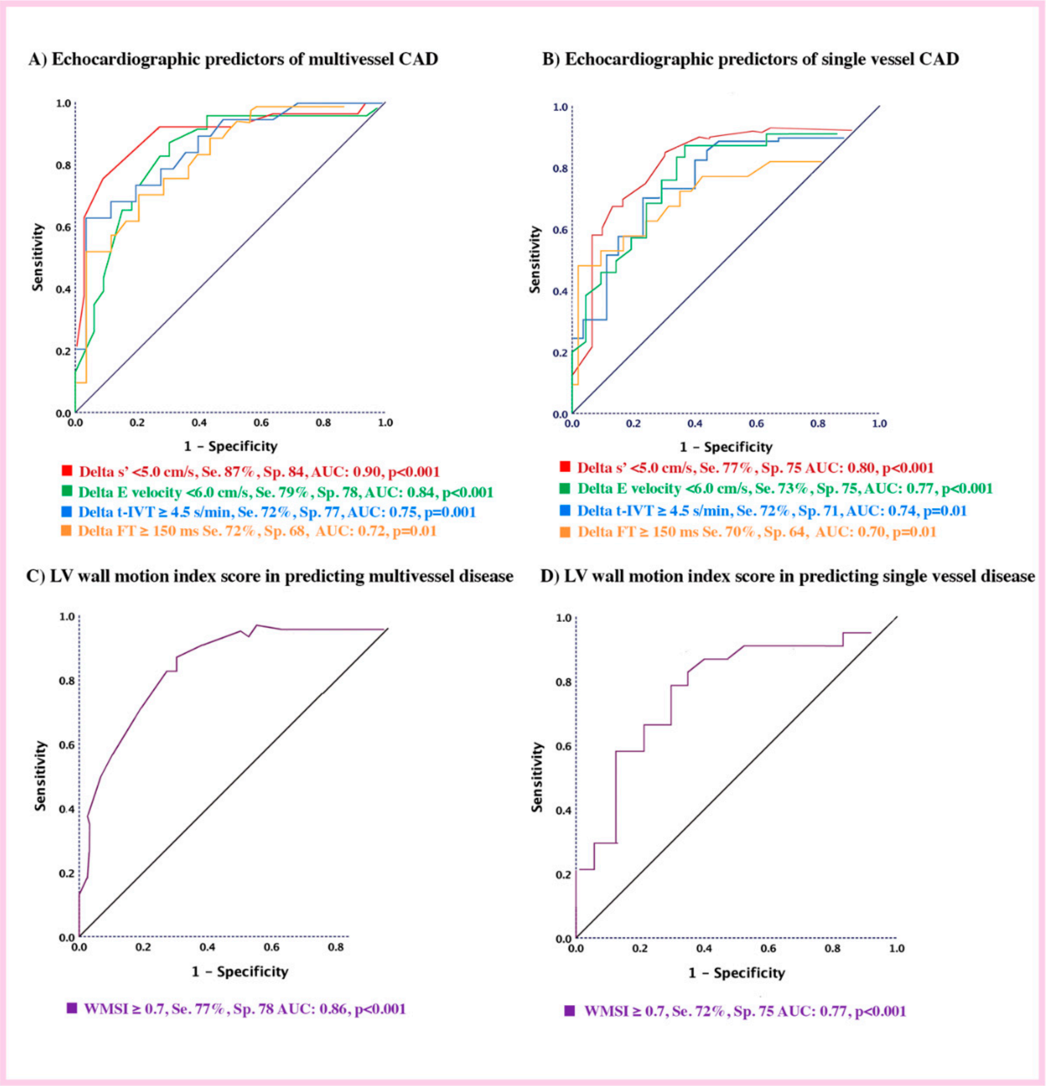

Figure S2. Echocardiographic predictors of multivessel and single vessel disease. **Figure S2.** Echocardiographic predictors of multivessel and single vessel disease. (**A**) Echocardiographic predictors of multivessel CAD. (**B**) Echocardiographic predictors of signal vessel CAD. (**C**) LV wall motion score index in predicting multivessel disease. (**D**) LV wall motion score index in predicting signal vessel disease.
